# A dual-role of SARS-CoV-2 nucleocapsid protein in regulating innate immune response

**DOI:** 10.1038/s41392-021-00742-w

**Published:** 2021-09-01

**Authors:** Yinghua Zhao, Liyan Sui, Ping Wu, Wenfang Wang, Zedong Wang, Yang Yu, Zhijun Hou, Guangyun Tan, Quan Liu, Guoqing Wang

**Affiliations:** 1 Center for Pathogen Biology and Infectious Diseases, Key Laboratory of Organ Regeneration and Transplantation of the Ministry of Education, The First Hospital of Jilin University, State Key Laboratory of Human-Animal Zoonotic infectious Diseases, Changchun, China; 2 College of Wildlife and Protected Area, Northeast Forestry University, Harbin, China; 3 Department of Pathogenbiology, The Key Laboratory of Zoonosis, Chinese Ministry of Education, College of Basic Medicine, Jilin University, Changchun, China; 4 Hospital of Stomatology, Jilin University, Changchun, China; 5 School of Life Sciences and Engineering, Foshan University, Foshan, China; 6 Institute of Animal Health, Guangdong Academy of Agricultural Sciences, Guangzhou, China

**Keywords:** Infection, Innate immunity

## Abstract

The recently emerged severe acute respiratory syndrome coronavirus 2 (SARS-CoV-2), which is the causative agent of ongoing global pandemic of COVID-19, may trigger immunosuppression in the early stage and overactive immune response in the late stage of infection; However, the underlying mechanisms are not well understood. Here we demonstrated that the SARS-CoV-2 nucleocapsid (N) protein dually regulated innate immune responses, i.e., the low-dose N protein suppressed type I interferon (IFN-I) signaling and inflammatory cytokines, whereas high-dose N protein promoted IFN-I signaling and inflammatory cytokines. Mechanistically, the SARS-CoV-2 N protein dually regulated the phosphorylation and nuclear translocation of IRF3, STAT1, and STAT2. Additionally, low-dose N protein combined with TRIM25 could suppress the ubiquitination and activation of retinoic acid-inducible gene I (RIG-I). Our findings revealed a regulatory mechanism of innate immune responses by the SARS-CoV-2 N protein, which would contribute to understanding the pathogenesis of SARS-CoV-2 and other SARS-like coronaviruses, and development of more effective strategies for controlling COVID-19.

## Introduction

Severe acute respiratory syndrome coronavirus 2 (SARS-CoV-2) is a new member in the *Coronaviridae* family that is genetically related to SARS-CoV and Middle East respiratory syndrome coronavirus (MERS-CoV).^1,2^ Hundreds millions of people have suffered from Coronavirus disease 2019 (COVID-19) caused by SARS-CoV-2 (https://covid19.who.int/). The elderly, especially those with comorbid lung disease, heart disease, diabetes, or obesity, have the most severe clinical symptoms, leading to a high mortality.^3^ SARS-CoV-2 triggers an immunosuppression in the early stage of infection, which contributes to uncontrolled coronaviral replication.^4–6^ This overwhelming coronaviral replication, in turn, stimulates an aberrant unchecked cytokine release, known as “cytokine storm”, resulting to severe acute respiratory distress syndrome (ARDS), pneumonia, and multiple organs failure.^7,8^ Understanding the underlying mechanisms is conducive to the development of more effective strategies for COVID-19.

Type I interferons (IFN-I) play critical roles in host antiviral process at the early stage of infection, IFN-I production is rapidly triggered by recognition of pathogen-associated molecular patterns (PAMPs) through pattern recognition receptors (PRRs).^9,10^ Like most RNA viruses, coronaviral RNA is recognized by retinoic acid-inducible gene I (RIG-I) and melanoma differentiation-associated gene 5 (MDA5), which activate various signaling pathways that finally lead to the production of IFN-I, interferon stimulated genes (ISGs), and proinflammatory cytokines.^11^ SARS-CoV-2 has employed several different mechanisms to escape the IFN response. For example, membrane (M) protein targets RIG-I/MDA-5 signaling, binds mitochondria antiviral signaling protein (MAVS) to impair its aggregation, and promotes TANK binding kinase 1 (TBK1) degradation via ubiquitination pathway;^12,13^ non-structural protein 1 (NSP1) binds the 40S ribosomal subunit to shutdown mRNA translation of IFNs and ISGs;^14^ NSP6 binds TBK1 to inhibit IRF3 phosphorylation;^15^ NSP13 binds TBK1 to block its phosphorylation;^15^ open-reading frame 6 (ORF6) binds importin karyopherin alpha 2 to inhibit IRF3 and STAT1 nuclear translocation;^15–17^ and ORF3b increases the IFN-antagonistic activity through its truncated C-terminal.^18^

In addition, SARS-CoV-2 induces substantial but delayed IFN production in the late stage of infection, and the coronaviral proteins exert divergent effects in the innate immune responses.^16^ SARS-CoV-2 nucleocapsid (2N) protein is a 419-amino acid structural protein that inhibits IFN-I signaling induced by SeV,^19^ and represses RIG-I mediated IFN-β production with transfected 2N plasmid at a low dose of 0.5 μg.^20^ However, SARS-CoV-2 N is also shown to promote RIG-I or MDA5-induced IFN-β activity and to facilitate hyperinflammation response.^16,21^ Here we demonstrated that SARS-CoV-2 N protein dually regulated IFN-I production, i.e., the low-dose 2N suppressed IFN-I production via sequestering TRIM25 to inhibit RIG-I ubiquitination, while high-dose 2N promoted IFN-I signaling. Additionally, SARS-CoV N protein was also found to bidirectionally regulate inflammatory cytokines expression. Our findings revealed an innate immune regulation mechanism mediated by the SARS-CoV-2 N protein, which would contribute to understanding the pathogenesis of SARS-CoV-2 N and SARS-like coronaviruses.

## Results

### N protein dually regulates IFN-I and inflammatory cytokines production

**Fig. 1:**
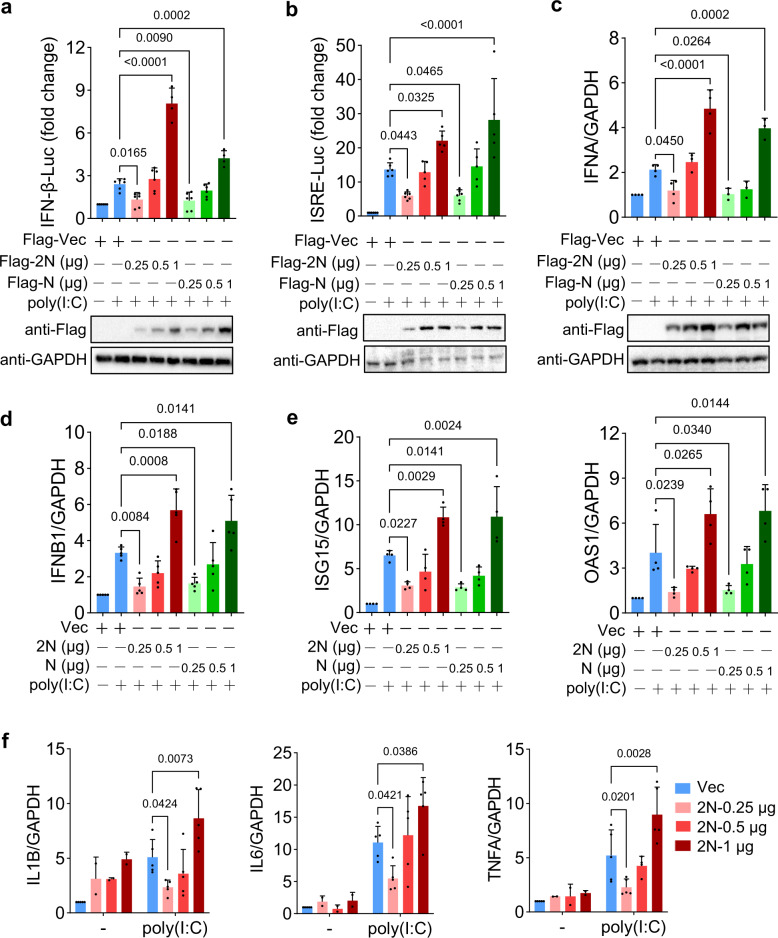
N proteins dually regulate production of IFN-I and inflammatory cytokines. **a**, **b** HEK293T cells in 24-well plate were co-transfected with an IFN-β promoter (**a**) or ISRE reporter plasmid (**b**), along with control plasmid pGL4.74 and 0.25, 0.5, or 1 μg SARS-CoV-2 or SARS-CoV N (2N or N) plasmids, then treated with or without poly(I:C), the empty vector as a control. At 24 h post-transfection (hpt), cells were harvested and luciferase activity was measured (upper); the expression of 2N and N proteins was detected by western blot, GAPDH as a loading control (down). **c**–**e** HEK293T cells in 24-well plate were transfected with 0.25, 0.5, or 1 μg 2N or N plasmids, then treated with or without poly(I:C). At 24 hpt, the mRNA expression of *IFNA* (**c**), *IFNB1* (**d**), and interferon-stimulated genes *ISG15* and *OAS1* (**e**) were examined using qPCR. **f** HEK293T cells in 24-well plate were transfected with a dose-gradient 2N plasmid, then treated with or without poly(I:C). At 24 hpt, the mRNA expression of *IL1B*, *IL6*, and *TNFA* were examined using qPCR. Results shown are the mean ± SD of at least three independent experiments

To explore the effect of SARS-CoV-2 N protein on IFN signaling, we constructed the expression plasmid of SARS-CoV-2 N (Supplementary Fig. S1) and examined its regulation of IFN-I production by dual luciferase reporter assay, in which SARS-CoV N was used as a control. The expression of SARS-CoV-2 N (2N) and SARS-CoV N (N) were confirmed by western blot (Fig. 1a–c down and Supplementary Fig. S2c). Unexpectedly, we found that low-dose (0.25 μg) 2N significantly reduced the promoter activity of IFN-β and interferon-stimulated response elements (ISRE) induced by poly(I:C) in HEK293T (Fig. 1a, b) and HepG2 (Supplementary Fig. S2a, b) cell lines, while high-dose (1 μg) 2N increased the promoter activity. Similar phenomenon was also observed in N transfected cells (Fig. 1a, b). We further confirmed the effects of 2N and N proteins on the transcription levels of IFN-I and ISGs. Quantitative real-time PCR (qPCR) showed that both low-dose (0.25 μg) N proteins significantly inhibited poly(I:C)-induced *IFNA*, *IFNB1*, *ISG15* (interferon-stimulated gene 15), *OAS1* (2′−5′-oligoadenylate synthetase 1), and *MxA* (MX dynamin like GTPase 1) expression, whereas both high-dose (1 μg) N proteins had the contrary effects (Fig. 1c–e and Supplementary Fig. S2d–f). We further identified the dually regulatory effect of 2N on IFN-I and ISGs production in A549 cells induced by the constitutively active N-terminal domains of RIG-I (RIG-IN) and MDA5 (Supplementary Fig. S3a–d). Moreover, 2N also dually regulated ISRE promoter activity induced by IFN-β in HEK293T (Supplementary Fig. S3e) and HepG2 (Supplementary Fig. S3f) cells, indicating that 2N could directly target the downstream signaling pathways of IFN to regulate ISGs expression. These results showed that low-dose SARS-CoV-2 and SARS-CoV N proteins suppress the expression of IFN-I and ISGs, while high-dose N proteins promote the IFN-I signaling.

Coronavirus infections evade innate immunity response during the first ten days, but the subsequent uncontrolled IFN-I response is associated with robust virus replication and severe complications, such as inflammation and “cytokine storm”.^5,7^ To determine whether the high-dose 2N promotes expression of disease-related inflammatory factors, such as interleukin 1 beta (IL1B), interleukin 6 (IL6), and tumor necrosis factor-alpha (TNFA),^8,22^ we transfected HEK293T cells with a dose-gradient 2N with or without poly(I:C) stimulation; qPCR assays showed that the mRNA levels of *IL1B*, *IL6*, and *TNFA* were all increased upon the high-dose 2N, and reduced upon the low-dose 2N, with a similar expression pattern of IFN-I production (Fig. 1f). In addition, we found that SARS-CoV N protein also bidirectionally regulated the expression of *IL1B*, *IL6*, and *TNFA* (Supplementary Fig. S2g). These results indicated that SARS-CoV-2 and SARS-CoV N proteins possess dually regulatory properties of the inflammatory cytokines.

### N protein dually regulates phosphorylation and nuclear translocation of IRF3, STAT1, and STAT2

The antiviral signal pathways mediated by IFN-I include two stages of IFN-I production and signal transduction. In the first stage, viral infection is rapidly sensed by host PRRs, and cascades the phosphorylation of interferon regulatory factor 3 (IRF3), followed by entering the nucleus to activate the expression of IFN-I.^23^ In the second stage, secreted IFN-α/β triggers the Janus kinases/signal transducer activator transcription proteins (JAK/STAT) signaling pathways. Phosphorylated STAT1 and STAT2 can form heterodimers, then combine with interferon regulatory factor 9 (IRF9) to form transcription complex IFN-stimulated gene factor 3 (ISGF3), which translocates to the nucleus to induce ISGs, and ultimately elicits an effective antiviral responses.^24^

**Fig. 2:**
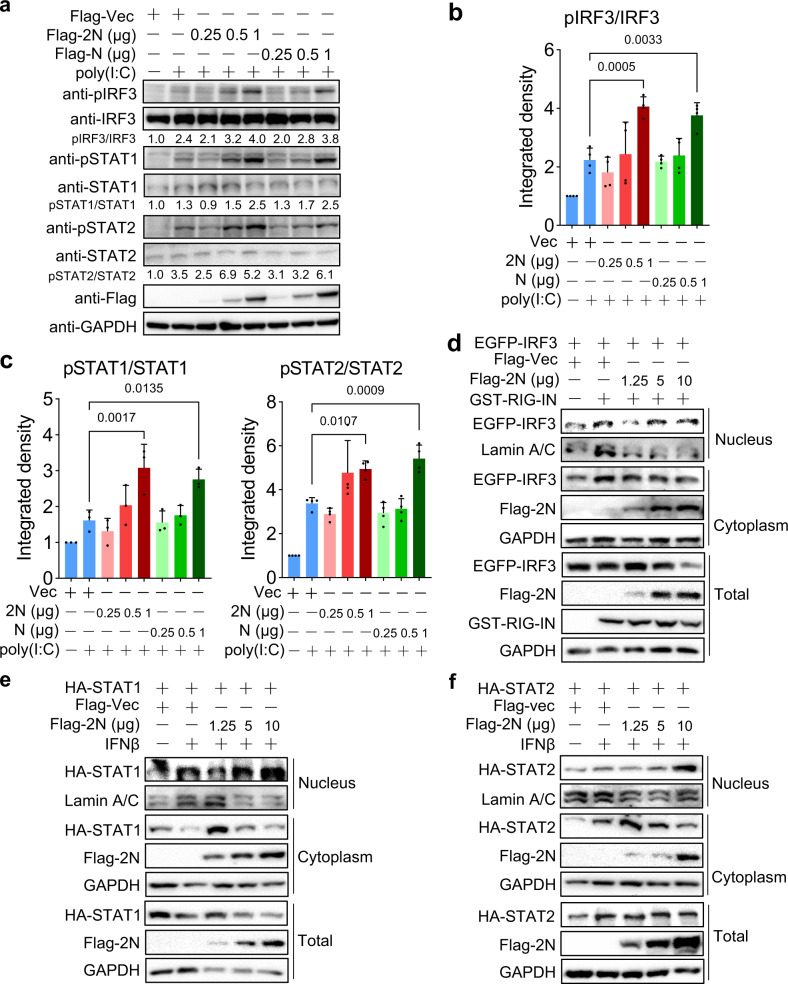
N proteins dually regulate phosphorylation and nuclear translocation of IRF3, STAT1, or STAT2. **a**–**c** HEK293T cells in 24-well plate were co-transfected with the plasmids expressing Flag-tagged SARS-CoV-2 or SARS-CoV N (2N or N) as indicated doses, then treated with or without poly(I:C). At 24 hpt, the expression of N proteins, the endogenous phosphorylation, and total proteins of IRF3, STAT1, or STAT2 were detected by western blot, GAPDH as a loading control (**a**); the gray-scale statistical analysis of phosphorylation levels of IRF3 (**b**) and STATs (**c**). **d**–**f** HEK293T cells in 6-cm plate were co-transfected with the plasmids as indicated. At 24 hpt, cells were treated with or without IFN-β for 30 min, and subjected to cell fractionation assay. Results shown are the mean ± SD of at least three independent experiments

To explore the mechanism of SARS-CoV-2 N protein-mediated dual regulation of IFN-I signaling, we tested the expression and activation of endogenous IRF3, STAT1 and STAT2 upon 2N protein. HEK293T cells were transfected with a dose-gradient SARS-CoV-2 and SARS-CoV N proteins, then activated with or without poly(I:C). Immunoblot indicated that ectopic expression of 2N and N proteins did not affect the total IRF3, STAT1, or STAT2, whereas the phosphorylation of IRF3 (Ser396), STAT1 (Tyr701), and STAT2 (Tyr689) was significantly increased by both N proteins in a dose-dependent manner (Fig. 2a–c). However, p-IRF3, p-STAT1, and p-STAT2 were slightly reduced upon both low-dose N proteins compared with poly(I:C)-activated vector control, probably due to a weak activation of IFN-I signaling by poly(I:C) in the study (Fig. 2a–c), but the decreasing trend was consistent with previous investigations.^19,20^ Additionally, 2N protein also regulated the endogenous phosphorylation of TBK1, IRF3, IKKε, and p65 in a similar pattern (Supplementary Fig. S3g).

**Fig. 3:**
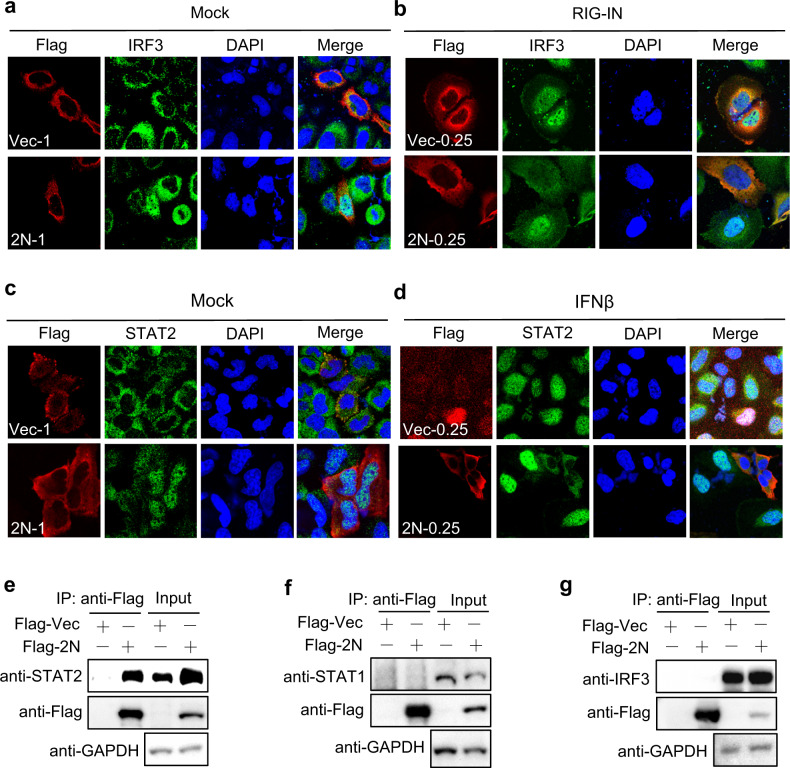
The interaction of 2N and IRF3, STAT1, or STAT2. **a** HepG2 cells were transfected with 1 μg Flag-tagged SARS-CoV-2 N (Flag-2N) or vector plasmids in 24-well plate. At 24 hpt, cells were subjected to immunofluorescence with anti-IRF3 and Flag antibodies. **b** HepG2 cells were transfected with 0.25 μg Flag-2N or vector together with RIG-IN. At 24 hpt, cells were subjected to immunofluorescence. Green: IRF3 signal; Red: 2N signal; Blue: DAPI (4, 6-diamino-2-phenyl indole, nuclei staining). Merge indicate the merged red, green, and blue channels. **c** HepG2 cells were transfected with 1 μg Flag-2N or vector plasmids. At 24 hpt, cells were subjected to immunofluorescence with anti-STAT2 and Flag antibodies. **d** HepG2 cells were transfected with 0.25 μg Flag-2N or vector for 24 h, then cells were treated with IFN-β for 30 min, and subjected to immunofluorescence. Green: STAT2 signal; Red: 2N signal; Blue: DAPI. **e-g** HEK293T cells were transfected with the plasmids as indicated. At 24 hpt, anti-Flag immunoprecipitates were analyzed by immunoblot with STAT2 (**e**), STAT1 (**f**) and IRF3 (**g**) antibodies

HEK293T cells were co-transfected with IRF3, STAT1, or STAT2 and different doses of 2N plasmids, then the cellular proteins were fractionated. The results showed that IRF3 was reduced in the nuclear fraction in the presence of low-dose 2N, while it was increased by the high-dose 2N induced with RIG-IN (Fig. 2d), and the nuclear translocation of STAT1 and STAT2 had the same trend with IFN-β induction (Fig. 2e, f). Furthermore, we transfected a high-dose (1 μg) 2N into HepG2 cells, immunofluorescence showed that high-dose 2N promoted endogenous IRF3 and STAT2 to enter nucleus compared with empty vector (Fig. 3a, c). In contrast, low-dose (0.25 μg) 2N significantly inhibited IRF3 and STAT2 nuclear translocation induced by RIG-IN or IFN-β (Fig. 3b, d). We also identified the regulatory effect of IRF3, STAT1, or STAT2 nuclear translocation by 2N and N proteins induced by poly(I:C) in HEK293T cells (Supplementary Fig. S4).

To further explore the interaction of 2N protein and IRF3, or STATs, HEK293T cells was transfected with 2N plasmid, and co-IP assays showed that 2N interacted with endogenous STAT2 (Fig. 3e), but not with IRF3 and STAT1 (Fig. 3f, g).

### 2N protein inhibits IFN-I production through TRIM25

**Fig. 4:**
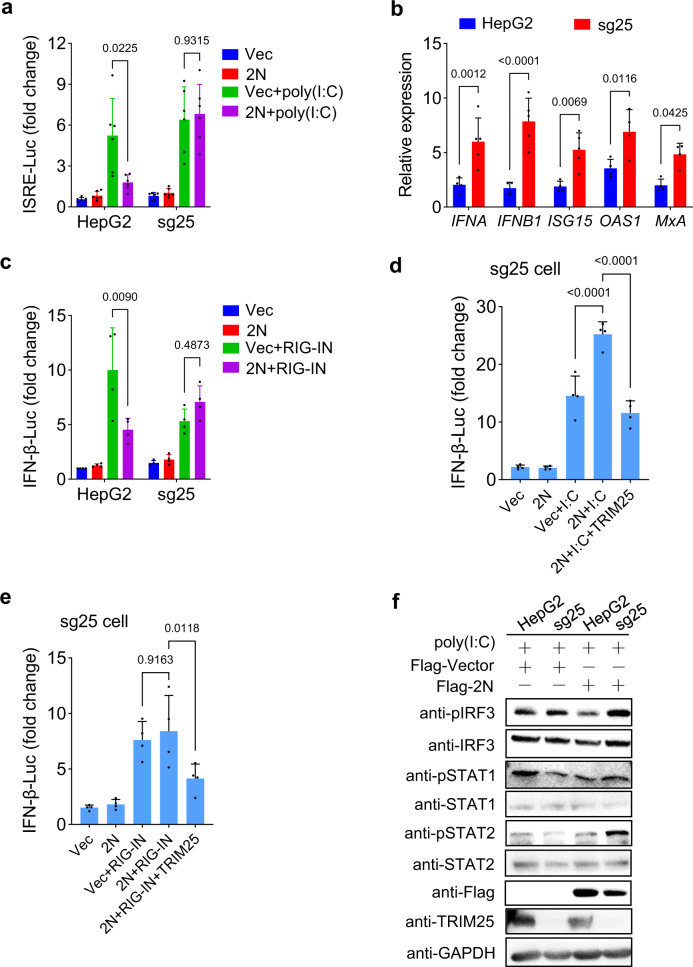
Low-dose 2N protein inhibits IFN-I production through TRIM25. **a** TRIM25 knockout (sg25) and WT HepG2 cells in 24-well plate were transfected with an ISRE reporter and 0.25 μg 2N plasmids, together with or without poly(I:C). At 24 hpt, cells were harvested and luciferase activity was measured. **b** sg25 and HepG2 cells in 24-well plate were transfected with 0.25 μg 2N and poly(I:C). At 24 hpt, the mRNA levels of *IFNA*, *IFNB1*, *ISG15*, *OAS1*, and *MxA* were determined using qPCR. **c** sg25 and HepG2 cells were transfected with the plasmids as indicated. At 24 hpt, the luciferase activity were measured. **d**, **e** sg25 cells were co-transfected with the plasmids as indicated. At 24 hpt, the luciferase activity was measured. **f** sg25 and HepG2 cells were transfected with 0.25 μg Flag-2N or empty vector together with poly(I:C). At 24 hpt, the phosphorylation levels of IRF3, STAT, or STAT2 were detected by western blot. Results shown are the mean ± SD of at least three independent experiments

The tripartite motif protein 25 (TRIM25) is an E3 ubiquitin ligase that participates in various cellular processes, including regulating the innate immunity against virus infections.^25–28^ In addition, TRIM25-mediated RIG-I ubiquitination and IFN induction is crucial in response to RNA virus infection.^29^ We found that the inhibition of IFN-I activity by 2N was rescued at transcription level through overexpression of TRIM25 (Supplementary Fig. S5a–c). We further examined the ability of low-dose 2N to inhibit IFN-I in TRIM25 knockout (sg25) cells. As expected, 2N failed to inhibit the IFN-β and ISRE promoter and transcriptional activation of IFN-I and ISGs in sg25 cells induced by poly(I:C) or RIG-IN (Fig. 4a–c), and ectopic expression of TRIM25 restored the inhibitory effect of 2N in sg25 cells (Fig. 4d, e). Whereas it is unexpected that poly(I:C) and RIG-IN induced the IFN-β and ISRE promoter activity in sg25 cells (Fig. 4a, c), which may be caused by the different function of endogenous and ectopic TRIM25.^29,30^ In addition, low-dose 2N increased phosphorylation of IRF3 and STATs in sg25 cells, also indicating that TRIM25 is responsible for the inhibitory effect of 2N on IFN-I signaling (Fig. 4f). Collectively, these data demonstrated that TRIM25 is involved in the inhibition of IFN-I signaling by low-dose 2N.

### 2N protein inhibits the interaction of TRIM25 and RIG-I

**Fig. 5:**
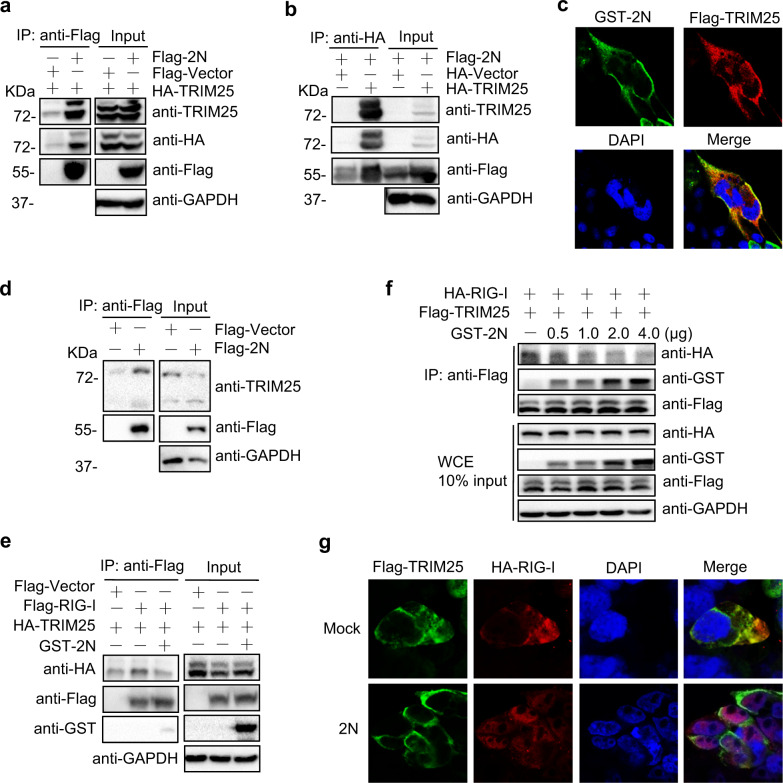
2N protein inhibits the interaction of TRIM25 and RIG-I through competitively binding to TRIM25. **a** HEK293T cells were transfected with the plasmids expressing 1 μg Flag-tagged SARS-CoV-2 N (Flag-2N) or empty-vector together with HA-TRIM25 in six-well plate. After 24 hpt, the cell lysates were subjected to anti-Flag immunoprecipitation (IP) and analyzed by immunoblot with anti-TRIM25, HA, and Flag antibodies, GAPDH was used as a loading control. **b** HEK293T cells were transfected with the indicated plasmids as in **a** for 24 h, the cell lysates were subjected to anti-HA IP and analyzed by immunoblot. **c** HEK293T cells were transfected with the plasmids expressing 0.25 μg GST-2N and Flag-TRIM25 in 24-well plate. After 24 hpt, the cells were subjected to immunofluorescence with anti-GST and Flag antibodies. **d** HEK293T cells were transfected with Flag-2N or empty vector for 24 h, the cell lysates were subjected to anti-Flag IP and analyzed by immunoblot with anti-TRIM25 and Flag antibodies. **e** HEK293T cells were transfected with Flag-RIG-I or empty vector and HA-TRIM25, together with or without 1 μg GST-2N plasmids in six-well plate. At 24 hpt, anti-Flag immunoprecipitates were analyzed by immunoblot. **f** HEK293T cells were transfected with Flag-TRIM25 and HA-RIG-I, together with an increasing concentration of GST-2N for 24 h in 6-well plate, anti-Flag immunoprecipitates were analyzed by immunoblot. **g** HEK293T cells were transfected with Flag-TRIM25 and HA-RIG-I, together with or without 0.25 μg 2N plasmids in 24-well plate. After 24 hpt, the transfected cells were subjected to immunofluorescence with anti-Flag and HA antibodies

Both SARS-CoV and MERS-CoV N proteins can interfere with the association of TRIM25 and RIG-I by interacting with TRIM25.^31,32^ To determine whether 2 N specifically binds to TRIM25, we co-transfected 2N and TRIM25 into HEK293T cells. After 24 h post-transfection, the cell lysates were subjected to immunoprecipitation (IP). We found that TRIM25 interacted with 2N reciprocally (Fig. 5a, b). Immunofluorescence also revealed that 2N was co-localized with TRIM25 (Fig. 5c). We further confirmed that 2N protein interacted with endogenous TRIM25 (Fig. 5d).

Then, we explored whether 2N protein inhibits the interaction of TRIM25 and RIG-I. The plasmids expressing TRIM25 and RIG-I, together with or without 2N, were co-transfected into HEK293T cells, and co-IP assays demonstrated that the interaction of TRIM25 with RIG-I was inhibited by 2N in a dose-dependent manner (Fig. 5e, f). Immunofluorescence also revealed that the co-localization of RIG-I and TRIM25 was destroyed by co-expression of 2N (Fig. 5g). Collectively, these findings demonstrated that SARS-CoV-2 N negatively regulates the interaction of TRIM25 and RIG-I.

### 2N protein inhibits RIG-I ubiquitination through TRIM25

**Fig. 6:**
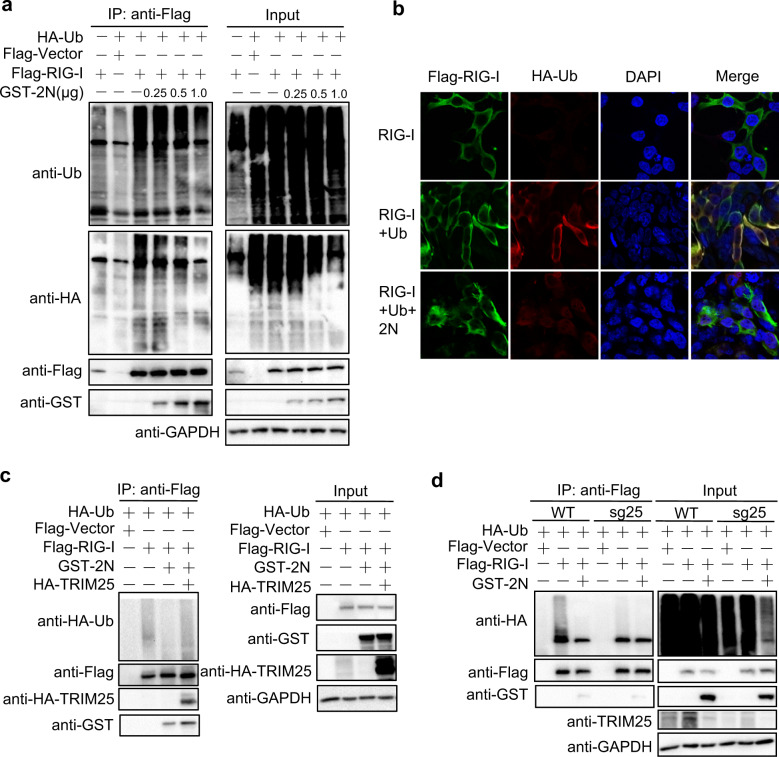
2N protein inhibits RIG-I ubiquitination through TRIM25. **a** HEK293T cells in six-well plate were transfected with HA-Ub and Flag-RIG-I plasmids as indicated, together with an increasing GST-SARS-CoV-2 N (GST-2N) plasmid. At 24 hpt, anti-Flag immunoprecipitates were analyzed by immunoblot with anti-Ub, HA, Flag, and GST antibodies. **b** HEK293T cells in 24-well plate were transfected with Flag-RIG-I, or Flag-RIG-I and HA-Ub, or Flag-RIG-I, HA-Ub, and 0.25 μg GST-2N plasmids. At 24 hpt, the cells were subjected to immunofluorescence with anti-Flag and HA antibodies. **c** HEK293T cells in six-well plate were transfected with the plasmids as indicated and 1 μg 2N plasmid, anti-Flag immunoprecipitates were analyzed by immunoblot. **d** sg25 and WT cells in six-well plate were transfected with the indicated plasmids and 1 μg 2N plasmid, anti-Flag immunoprecipitates were analyzed by immunoblot

TRIM25 is involved in the K63-linked ubiquitination of RIG-I, which is essential for IFN-I production.^29,33^ We next investigated the role of 2N protein in the TRIM25-mediated ubiquitination of RIG-I. First, we examined whether 2N inhibits RIG-I ubiquitination, HEK293T cells were transfected with RIG-I and 2N, together with or without ubiquitin plasmids. The results indicated that ubiquitination of both exogenous and endogenous RIG-I was significantly impaired by 2N protein in a dose-dependent manner (Figs. 6a and Supplementary Fig. S5d), which was also confirmed by immunofluorescence (Fig. 6b). We further found that overexpression of TRIM25 could rescue the suppression of RIG-I ubiquitination caused by 2N and RIG-I interacted with 2N (Fig. 6c). Furthermore, the RIG-I ubiquitination was reduced in sg25 cells, and the inhibition of RIG-I ubiquitination by 2N was also abolished (Fig. 6d). These results indicated that SARS-CoV-2 N inhibits RIG-I ubiquitination through interfering with the interaction of TRIM25 and RIG-I.

### Interaction domains of 2N protein and TRIM25

**Fig. 7:**
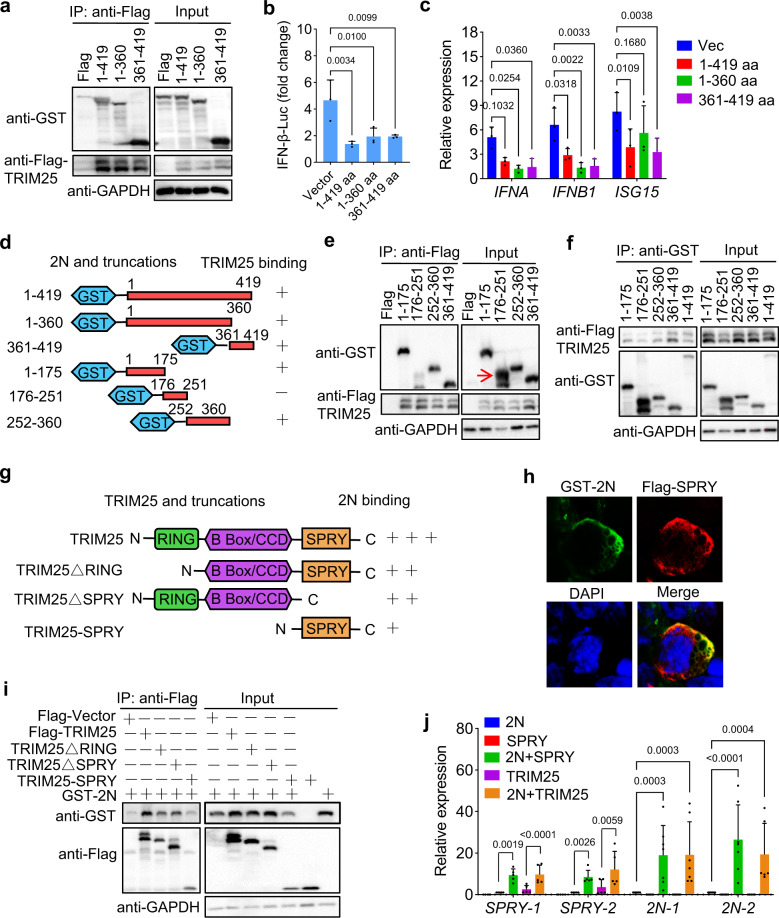
The interaction domains of 2N protein and TRIM25. **a** HEK293T cells were transfected with the plasmids expressing GST-tagged full-length or truncated SARS-CoV-2 N (2N) protein, together with Flag-TRIM25 or Flag-vector. At 24 hpt, anti-Flag immunoprecipitates were analyzed by immunoblot with anti-GST and Flag antibodies. **b**, **c** HEK293T cells were transfected with an IFN-β promoter reporter and the full-length or truncated 2N plasmids, then treated with poly (I:C). At 24 hpt, luciferase activity was measured (**b**), and the mRNA expression of *IFNA*, *IFNB1*, and *ISG15* were examined using qPCR (**c**). **d** Domain mapping of the SARS-CoV-2 N and TRIM25 association. **e** HEK293T cells were transfected with the indicated GST-tagged truncations of 2N and Flag-TRIM25 or Flag-vector. At 24 hpt, anti-Flag immunoprecipitates were analyzed by immunoblot. The arrow indicates the target protein band. **f** HEK293T cells were transfected with the GST-tagged truncations of 2N and Flag-TRIM25. At 24 hpt, the cell lysates were incubated using Glutathione Agarose to purify GST-S-2-N truncations. The purified proteins were analyzed by immunoblot. **g** Domain mapping of the association. **h** HEK293T cells were transfected with plasmids expressing GST-2N and Flag-TRIM25-SPRY domain. At 24 hpt, the cells were subjected to immunofluorescence analyses with anti-GST and Flag antibodies. **i** HEK293T cells were transfected with the plasmids expressing Flag-tagged full-length or truncated TRIM25, together with GST-2N. At 24 hpt, anti-Flag immunoprecipitates were analyzed by immunoblot. **j** HEK293T cells were transfected with the indicated plasmids. At 24 hpt, qPCR examined mRNA expression of SPRY and 2N. Results shown are the mean ± SD of at least three independent experiments

Specific functional domain of SARS-CoV N is responsible for suppression of IFN-I production.^32^ We sought to investigate which domain of SARS-CoV-2 N is involved in its interaction with TRIM25. Full-length or truncated fragments (aa 1 to 360 and 361 to 419) of 2N were co-transfected with TRIM25 into 293T cells. We found that both truncated proteins could bind to TRIM25 (Fig. 7a), and were sufficient to suppress IFN-I production (Fig. 7b, c). To determine the key domains for the truncated protein of 1–360 aa, we then constructed a series of truncations (Fig. 7d). The protein–protein interactions of these truncations with TRIM25 were examined via co-IP, demonstrating that the truncations (aa 1–175 and 252–360) of 2N could interact with TRIM25, but the truncation of aa 176–251 showed negative interaction (Fig. 7e, f).

As a ubiquitin E3 ligase, TRIM25 includes a RING-finger domain, a SPRY domain, a coiled-coil dimerization domain, and two B-box domains.^29^ Reciprocally, plasmids expressing SPRY, SPRY deletion, and RING-finger deletion truncations were constructed (Fig. 7g). We found that 2N was co-localized with SPRY domain of TRIM25, which was the same as SARS-CoV N (Figs. 7h and Supplementary Fig. S6d).^32^ Besides, co-IP showed that 2N can interact with both the SPRY- and RING-deleted TRIM25 truncations with the lower binding level than that of full-length TRIM25 (Figs. 7i and Supplementary Fig. S6a, b), indicating that 2N can interact with the SPRY and other domains of TRIM25, including the RING-finger domain, while only SPRY domain of TRIM25 is responsible for the interaction with SARS-CoV N (Supplementary Fig. S6c–e). These findings clearly defined the interaction domains of the SARS-CoV-2 N and TRIM25 (Fig. 7d, g).

We unexpectedly found that the expression of both 2N and SPRY domain was obviously reduced in the co-transfected cells (Figs. 7i and Supplementary Fig. S6a, b), while their mRNA levels were increased (Fig. 7j). Unlike inhibition of TRIM25-induced IFN-I production, 2N protein enhanced SPRY domain-induced IFN-I (Supplementary Fig. S7a), suggesting that 2N and SPRY may be degraded by post-transcriptional modifications after activation of IFN-I signaling. The ubiquitin-mediated degradation of the 2N and SPRY domain has been excluded in this research (Supplementary Fig. S7b–d), and the precise mechanism needs further investigation.

### SARS-CoV-2 induces excessive IFN-I production in late stage of infection

**Fig. 8:**
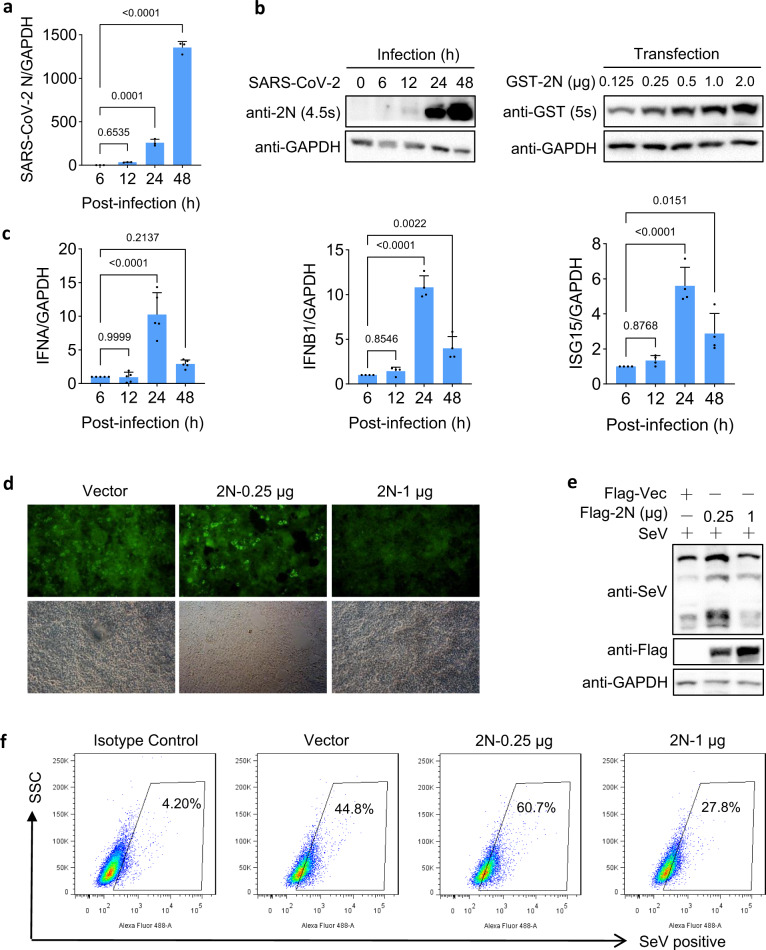
SARS-CoV-2 N protein dually regulates viral replication. **a**, **c** Caco-2 cells were infected with SARS-CoV-2 at MOI of 0.01. At 6, 12, 24, and 48 hpi, total RNA was extracted. The mRNA expression of the viral N gene (**a**) and host IFNA, IFNB1, ISG15 (**c**) relative to GAPDH control were examined using qPCR. **b** Caco-2 cells were infected with SARS-CoV-2 as indicated as in **a**, HEK293T cells were transfected with the indicated 2N plasmids. At 24 hpt, and lysis of infected and transfected cells (50 μg/sample) were analyzed by immunoblot. **d**–**f** The HEK293T cells were transfected with 2N plasmids as indicated. The cells were infected with SeV at 24 hpt. After 20 h, cells were analyzed with immunofluorescence (Scale bar, 100 μm) (**d**), immunoblot (**e**), and flow cytometry (**f**). Results shown are the mean ± SD of at least three independent experiments

COVID-19 patients have undergone an insufficient production of IFN-I in the early stage and cytokine storm in the late stage of infection.^6,7^ To understand the interaction of SARS-CoV-2 and the IFN-I signaling in vitro, Caco-2 cells were infected with the virus at MOI of 0.01. The cells were harvested at different time points post-infection and the timely expression of 2N was determined by qPCR and immunoblot, showing that 2N was significantly elevated at 24 and 48 h post-infection (hpi) (Fig. 8a, b). The expression of 2N in SARS-CoV-2 infected cells was comparable with its expression in transfected cells (Figs. 8b and Supplementary Fig. S10), indicating that 2N protein in transfected cells could reflect the physiological changes of viral infection. The expressions of *IFNA*, *IFNB1*, and *ISG15* was slightly increased before 12 hpi, and dramatically elevated at 24 hpi, while decreased at 48 hpi (Fig. 8c). In contrast, Sendai virus (SeV) infection induced the expression of *IFNB1* and *ISG56* as early as 6 hpi (Supplementary Fig. S9). These results showed that SARS-CoV-2 infection stimulates substantial but delayed IFN-I signaling, with a similar expression pattern in 2N transfected cells.

### 2N protein dually regulates SeV replication

To validate whether the SARS-CoV-2 N protein affects SeV replication, HEK293T cells expressing different doses of 2N or empty vector were infected with SeV, and the viral replication was determined by immunofluorescence, immunoblotting, and flow cytometry at 20 hpi. All of the results showed that low-dose (0.25 μg) 2N significantly promoted SeV replication, whereas high-dose (1 μg) 2 N inhibited SeV replication (Fig. 8d-f).

## Discussion

**Fig. 9:**
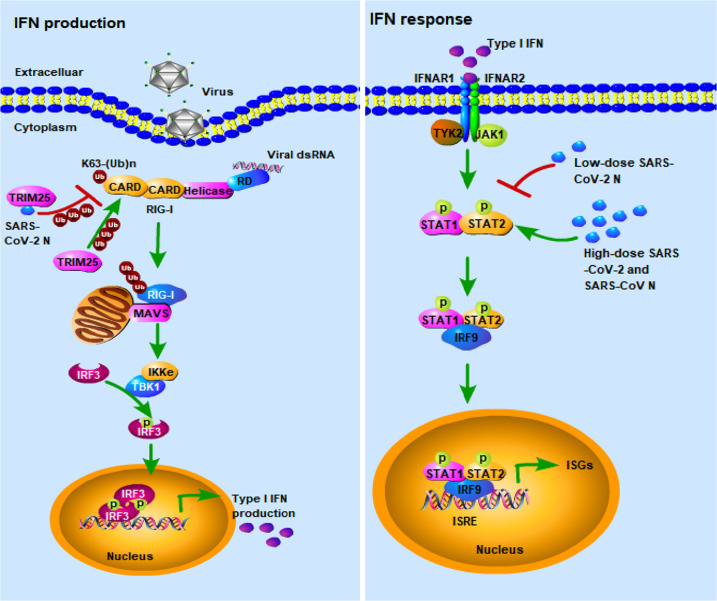
Schematic depiction of the dual regulation role of SARS-CoV-2 nucleocapsid protein against IFN-I signaling. The low-dose SARS-CoV-2 N protein suppresses innate immune response by reducing RIG-I ubiquitination through interacting with TRIM25, and reducing STAT1/STAT2 phosphorylation and nuclear translocation; whereas high-dose SARS-CoV-2 and SARS-CoV N proteins promote innate immune response by enhancing the phosphorylation and nuclear translocation of STAT1 and STAT2

COVID-19 patients have undergone a unique immune response, which embodies in the insufficient IFN-I production (immunosuppression) in the early stage of infection and cytokine storm (overactive immune response) in the late stage of infection.^6,34^ The two stages of immune reactions are associated with the severity of COVID-19. SARS-CoV-2 infection induces substantial but delayed IFN-β production in vitro.^16^ Understanding the underlying mechanisms would contribute to development of effective therapies for this emerging coronaviral disease. In this study, we demonstrated that low-dose SARS-CoV-2 N protein inhibits IFN-I production by disturbing the interaction of TRIM25 and RIG-I and inhibiting the phosphorylation and nuclear translocation of IRF3, STAT1 and STAT2, whereas high-dose N protein promotes the IFN-I and inflammatory cytokine expression by enhancing the phosphorylation and nuclear translocation of STAT1 and STAT2 (Fig. 9).

The severity of COVID-19 is positively correlated with the cytokine storm, which is characterized by the clinical manifestations of systemic inflammation, hemodynamic instability, hyperferritinemia, and multi-organ failure.^35–37^ Moreover, both SARS-CoV and MERS-CoV infections also trigger the cytokine storm that may contribute to occurrence of ARDS, the leading cause of death.^38,39^ The proinflammatory cytokines IL1B, IL6 and TNFA are the key components of cytokine storm during the coronavirus infections.^40,41^ But the mechanism by which coronavirus causes a cytokine storm is still not completely clear. Our study showed high-dose (1 μg) SARS-CoV-2 N protein could promote the expression of proinflammatory cytokines IL6, IL1B, and TNFA. Recent research has shown that SARS-CoV-2 N protein promotes the NACHT, LRR, and PYD domains-containing protein 3 (NLRP3) inflammasome activation to induce inflammatory response, which may explain the mechanism of N protein in promoting the production of IFN-I and inflammatory cytokines.^21^

During the virus infection, cGAS and RIG-I are two major sensors in the recognition of viral nucleic acids, which further lead to the induction of IFN. TRIM family members, including TRIM14, TRIM25, TRIM31 and TRIM38, are reported to positively regulate the IFN signaling.^29,42–44^ TRIM14 inhibits cGAS degradation and TRIM38 targets cGAS for sumoylation in response to DNA virus infection;^42,44^ TRIM31 promotes MAVS aggregation and activation through Lys63-linked polyubiquitination;^43^ and TRIM25-mediated RIG-I ubiquitination is crucial for the IFN production in RNA virus infection.^29^ SARS-CoV-2 is a positive single-stranded RNA virus, and MAVS is the downstream of RIG-I. Therefore, we first examined the role of TRIM25 in the SARS-CoV-2 N-mediated inhibition of IFN signaling, and found that low-dose 2N protein inhibited RIG-I ubiquitination through interacting with TRIM25. Accidentally, we also found poly(I:C) and RIG-IN induced the IFN-β and ISRE promoter activity in sg25 cells. Cadena et al.^30^ has demonstrated that TRIM25 is not the ubiquitin E3 ligase for RIG-I in multiple TRIM25-deficient cell lines, which may reflect the function of endogenous TRIM25. In contrast, TRIM25 is suggested to be an essential E3 ligases for RIG-I-mediated IFN-I production used ectopic expression of TRIM25 in several other studies.^29,32^ These confusing results may be caused by the different levels of endogenous and exogenous TRIM25. Moreover, the regulatory effect of 2N on IFN signaling in sg25 cells was exactly opposite to that in WT cells (Supplementary Fig. S8), indicating that high-dose 2N activates IFN-I through TRIM25. However, the two-way regulation mechanisms of TRIM25 in IFN signaling need further investigation.

SARS-CoV N protein has also been shown to inhibit IFN-I production by interfering with TRIM25.^32^ However, the domains of N proteins and TRIM25 involved in the inhibition and interaction are different in the two coronaviruses. We demonstrated that both N-terminal (aa 1–360) and C-terminal (aa 361–419) of 2N could suppress IFN-I production, while another report showed that only the N-terminal (aa 1–361) has inhibitory effect.^19^ Interestingly, only C-terminal (aa 362–422) of SARS-CoV N is responsible for the suppression of IFN-I production.^32^ We also found that there were multiple loci in 2N protein, including the N-terminal (aa 1–360 and aa 1–175), C-terminal (aa 361–419), and intermediate region (aa 252–360), that could bind to TRIM25, and 2N could interact with multiple domains of TRIM25, such as SPRY and RING-finger. Therefore, there are multiple loci in both 2N and TRIM25 involved in their interactions to inhibit IFN-I signaling. On the contrary, only single domain of SARS-CoV N protein C-terminus (aa 362–422) and TRIM25 SPRY domain is involved in their interaction to inhibit IFN-I signaling.^32^ These discrepancies may contribute to understanding the different pathogenicity of SARS-CoV and SARS-CoV-2.

In summary, we demonstrated that SARS-CoV-2 N protein dually regulates innate immune responses, in which low-dose N protein suppresses type I interferon (IFN-I) signaling and inflammatory cytokines, whereas high-dose N promotes IFN-I and inflammatory factors expression. Our findings reveal a regulatory mechanism of innate immune responses mediated by the SARS-CoV-2 N protein, which may contribute to development of more effective strategies for controlling COVID-19 and understanding the pathogenesis of SARS-CoV-2 and other SARS-like coronaviruses.

## Materials and methods

### Cells and antibodies

Human embryonic kidney cell HEK293T, hepatic carcinoma cell HepG2, human lung carcinoma cell A549, and human colorectal adenocarcinoma Caco-2 were maintained in Dulbecco’s modified Eagle’s medium (DMEM) (HyClone, Logan, UT) containing 10% inactivated fetal bovine serum (BBI, Shanghai, China), penicillin (100 IU/ml), and streptomycin (100 mg/ml) at 37 °C in a 5% CO_2_ atmosphere. Sg25 cell is derived from HepG2 cell, in which TRIM25 has been knocked out through the CRISPR/Cas9 system.^45^

Antibodies against STAT1, p-STAT1, and STAT2 were purchased from Abcam (Cambridge, MA, USA); anti-IRF3, anti-TBK1, anti-HA, anti-GST, anti-Flag, anti-GAPDH, CoraLite 594-conjugated IgG, and CoraLite 488-conjugated IgG secondary antibodies were obtained from Proteintech (Rosemont, IL, USA); anti-TRIM25, anti-IKKε, anti-pIKKε, anti-pTBK1, and anti-pIRF3 antibodies were from Cell Signaling technology (Danvers, MA, USA); anti-pSTAT2 antibody was from Sigma-Aldrich (St. Louis, MO, USA); anti-Lamin A/C antibody was from TransGen (Beijing, China); anti-SeV antibody was from MBL (Beijing, China); Human anti-SARS-CoV & CoV-2 NP antibody was from ZENBIO (Chengdu, China).

### Plasmid construction

The plasmids of IFN-β luciferase reporter, ISRE luciferase reporter, control reporter (pGL4.74), Flag-TRIM25, HA-TRIM25, and HA-ubiquitin were obtained as previously described.^45,46^ HA-tagged RIG-I plasmid was purchased from Public Protein/Plasmid Library (PPL, Jiangsu, China). GFP-tagged IRF3 and HA-tagged STAT1/2 plasmids were purchased from Miaoling Plasmid Library (Wuhan, China). Flag or GST-tagged RIG-I, SARS-CoV N protein (SARS coronavirus Tor2, NC_004718.3), SARS-CoV-2 N protein (IPBCAMS-WH-01/2019 strain, no. EPI_ISL_402123) and truncations were cloned into VR1012-based expression vector and confirmed by sequencing. The SARS-CoV-2 N protein shares 90.52% aa identity with the SARS-CoV N protein (Supplementary Fig. S1).

### Luciferase reporter assay

The luciferase reporter assay were performed as previously described.^47^ Briefly, HEK293T, A549, HepG2, or sg25 cells were transfected with a control plasmid or protein expression plasmids together with the luciferase reporter plasmids using Viafect TM Transfection Reagent (Promega, Madison, WI, USA) or Lipofectamine TM 2000 (Invitrogen, San Diego, CA, USA). After 8 h, the cells were then transfected with poly(I:C) (Invitrogen), and the promoter activity was measured with Dual-Luciferase® Reporter Assay System (Promega) 16 h later. The relative firefly luciferase activities are normalized to the Renilla luciferase.

### Quantitative real-time PCR

Total RNA was extracted using an EasyPure® RNA Kit (TransGen, Beijing, China), and the first-strand cDNA was synthesized by TransScript First-Strand cDNA Synthesis Super Mix (TransGen). The qPCR was analyzed with SYBR Green Master (Roche, Basel, Switzerland) as previously described.^47^ The results were normalized by the house-keeping gene GAPDH. Primers used for these analyses are listed in Supplementary Table S1.

### Coimmunoprecipitation and immunoblot analysis

Coimmunoprecipitation and immunoblot analyses were performed as previously described.^46^ In brief, cells were lysed in lysis buffer (50 mM Tris-HCl, pH 8.0, 150 mM NaCl, 1% NP-40) containing protease and phosphatase inhibitor cocktail (Selleck, Houston, Texas, USA). For coimmunoprecipitation, lysates were incubated overnight with ANTI-FLAG^®^ M2 Affinity Gel (Sigma-Aldrich), EZview™ Red Anti-HA Affinity Gel (Millipore, Billerica, MA, USA), or Pierce Glutathione Agarose (Thermo Scientific, Rockford, IL, USA), then proteins were separated by SDS-PAGE and transferred onto PVDF membranes. After blocking in TBST containing 5% BSA, the blots were probed with primary antibodies. Determination of the band intensities were performed with ChemiDoc XRS^+^ Molecular Imager software (Bio-Rad, Philadelphia, PA, USA).

### Immunofluorescence

HEK293T or HepG2 cells cultured on 12-mm coverslips were transfected with indicated plasmids. After 24 h, cells were fixed with 4% paraformaldehyde, and permeated with 0.5% Triton X-100. After cells were washed with PBST, they were blocked in 1% BSA and stained with primary antibodies, followed by staining with CoraLite 594- or CoraLite 488-conjugated IgG secondary antibodies.^48^ Nuclei were stained with DAPI (Yesen Biotechnology, Shanghai, China). Fluorescence images were obtained and analyzed using a confocal microscope (FV3000, OLYMPUS).

### Nuclear and cytoplasmic extraction

To prepare the nuclear and cytoplasmic fraction, cells were treated using the nuclear and cytoplasmic protein extraction kit (Beyotime, China) according to the manufacturer’s instructions. The purified cytoplasmic and nuclear fraction were subjected to Western blot assay according to the standard procedures with the relevant antibodies.

### Flow cytometry

Flow cytometry analyses were performed as previously described.^47^ In brief, cells were harvested, and intracellular staining was performed using a Cytofix/Cytoperm kit (BD Biosciences) according to the manufacturer’s instruction. After staining with an anti-SeV antibody, cells were analyzed using a BD LSRFortessa flow cytometer. Data analysis was carried out with the FlowJo software.

### Virus infection

SeV, kindly provided by Dr. Haoran Guo at the First Hospital of Jilin University, was used to infect HEK293T cells. In brief, SeV was diluted with serum-free DMEM and incubated with the target cells for 1 h, then change the serum-free medium to DMEM containing 10% FBS. SARS-CoV-2 (BetaCoV/Beijing/IME-BJ05/2020) was isolated from a patient with viral pneumonia in Wuhan, China. Caco-2 cells were infected with SARS-CoV-2 at a multiplicities of infection (MOI) of 0.01 and harvested at 6, 12, 24, and 48 h post infection. All experiments involving infectious SARS-CoV-2 were performed in biosafety level 3 (BSL3) laboratory in Academy of Military Medical Sciences (AMMS).

### Statistical analysis

The results are representative of at least three independent experiments and shown as the mean ± SD values. For statistical analysis, two-tailed unpaired Student’s *t* tests were performed in GraphPad Prism 9.0.2, and *P* value of <0.05 was considered statistically significant.

## Supplementary information

Supplementary material

## Data Availability

All data supporting the findings of this study are available within the article and its supplementary information or from the corresponding author upon reasonable request.
