# Supplementary material for: A dual-role of SARS-CoV-2 nucleocapsid protein in regulating innate immune response
Source: Signal Transduct Target Ther. 2021 Sep 1;6:331. doi: 10.1038/s41392-021-00742-w (PMC8409078; doi:10.1038/s41392-021-00742-w)
Supplement: Supplementary file 1 — Supplementary material [file 41392_2021_742_MOESM1_ESM.docx]

Supplementary Materials for

**A dual-role of SARS-CoV-2 nucleocapsid protein in regulating innate immune response**

Yinghua Zhao, Liyan Sui, Ping Wu, Wenfang Wang, Zedong Wang, Yang Yu, Zhijun Hou, Guangyun Tan*, Quan Liu*, Guoqing Wang*

Correspondence to: Guangyun Tan (tgy0425@jlu.edu.cn) or Quan Liu (liuquan1973@hotmail.com) or Guoqing Wang (qing@jlu.edu.cn)

**This PDF file includes:**

Figures. S1 to S10

Tables S1

Figure. S1.


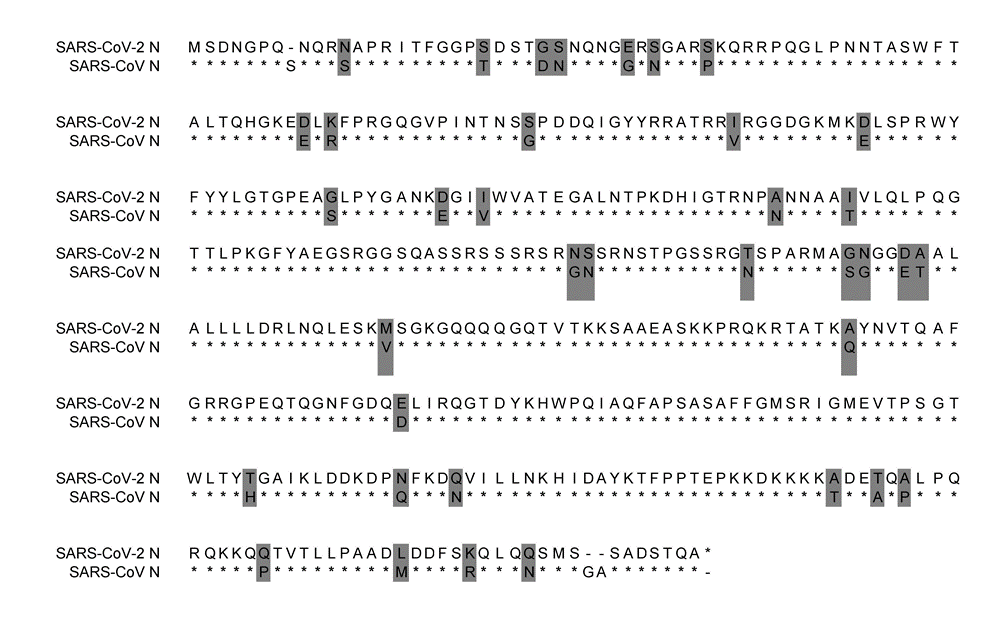


**Supplementary Figure 1. Amino acid identity between SARS-CoV-2 and SARS-CoV nucleocapsid proteins.** Amino acid alignment of SARS-CoV-2 and SARS-CoV nucleocapsid used in this study.

Figure. S2.

**
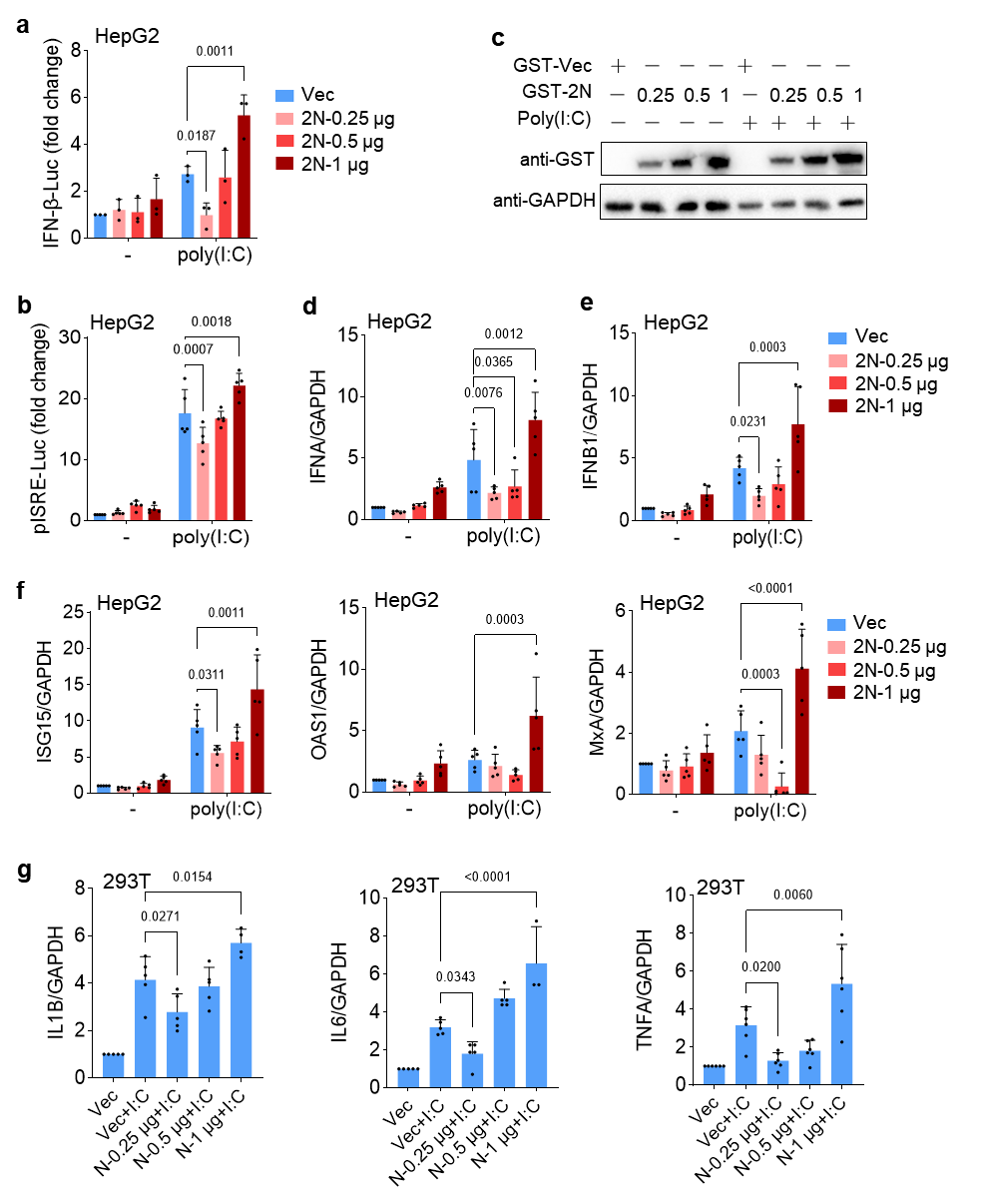
**

**Supplementary Figure 2. N proteins dually regulates IFN-I and inflammatory cytokine expression. a, b** HepG2 cells were co-transfected with an IFN-β promoter (**a**) or ISRE reporter plasmid (**b**), along with control plasmid pGL4.74 and SARS-CoV-2 N (2N) plasmids as indicated, then treated with or without poly(I:C), the empty vector as a control. At 24 hpt, cells were harvested and luciferase activity was measured. **c** The expression of 2N protein in (**a**) was detected by Western blot, GAPDH as a loading control. **d-f** HepG2 cells were transfected with 2N plasmid as indicated, then treated with or without poly(I:C). At 24 hpt, the mRNA expression of *IFNA* (**d**), *IFNB1* (**e**), and interferon stimulated genes *ISG15*, *OAS1* and *MxA* (**f**) were examined using qPCR. **g** HEK293T cells were transfected with the dose gradient SARS-CoV N plasmid, then treated with or without poly(I:C). At 24 hpt, the mRNA expression of *IL1B*, *IL6* and *TNFA* were examined using qPCR. Results shown are the mean ± SD of at least three independent experiments.

Figure. S3.

**
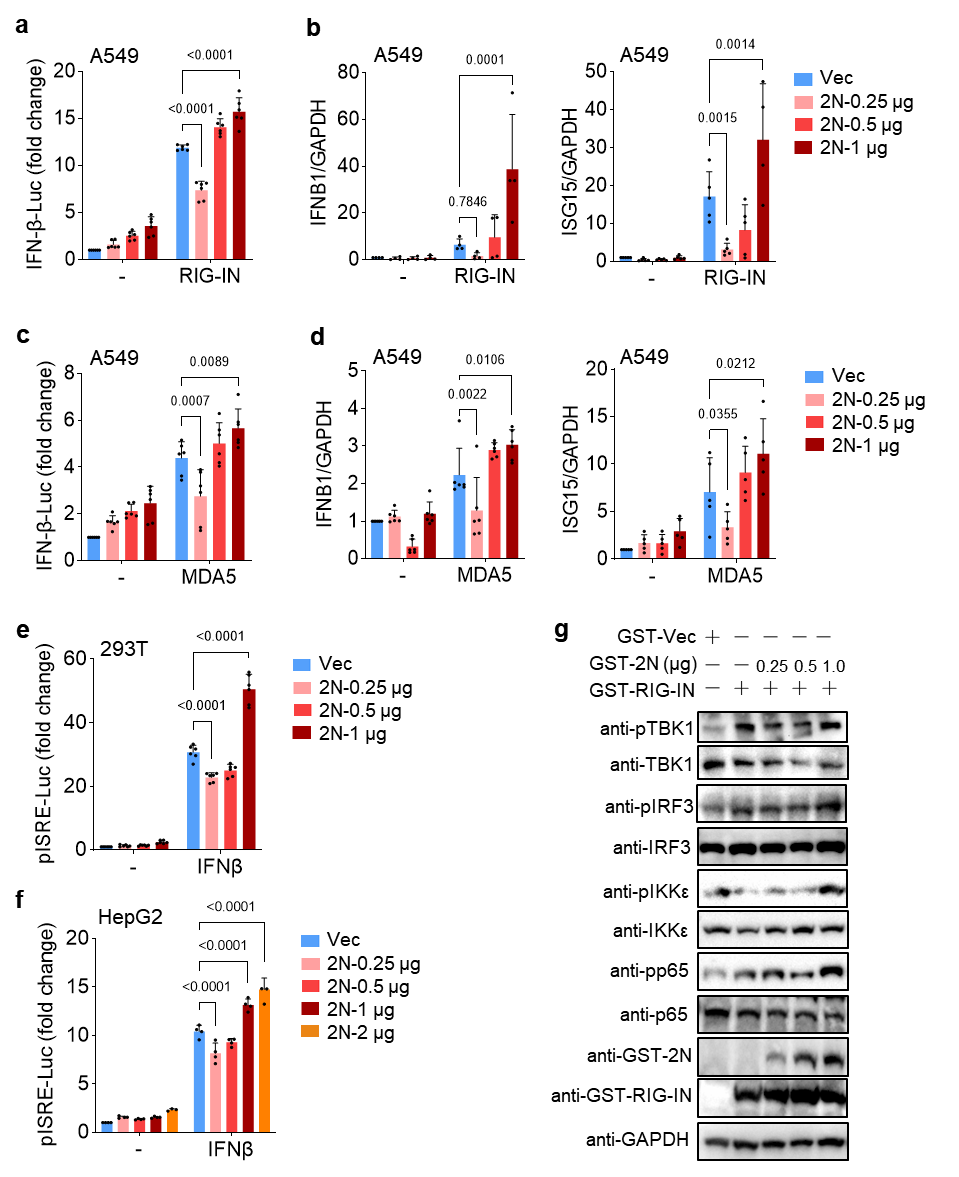
**

**Supplementary Figure 3. 2N protein dually regulates IFN-I signaling. a-d** A549 cells were co-transfected with an IFN-β promoter and SARS-CoV-2 N (2N) plasmid as indicated, then transfected with or without RIG-IN (**a**, **b**) and MDA5 (**c**, **d**). At 24 hpt, the luciferase activity were measured (**a**, **c**) and genes expression were analyzed by qPCR (**b**, **d**). **e, f** HEK293T (**e**) and HepG2 (**f**) cells were co-transfected with an ISRE promoter and 2N plasmid as indicated. At 24 hpt, cells were treated with or without IFN-β for 30 min, and the luciferase activity was measured. **g** A549 cells were co-transfected with the plasmids as indicated. At 20 hpt, the expression of 2N protein, the endogenous phosphorylation and total protein levels of TBK1, IRF3, IKKε and p65 were detected by Western blot, GAPDH as a loading control.

Figure. S4.

**
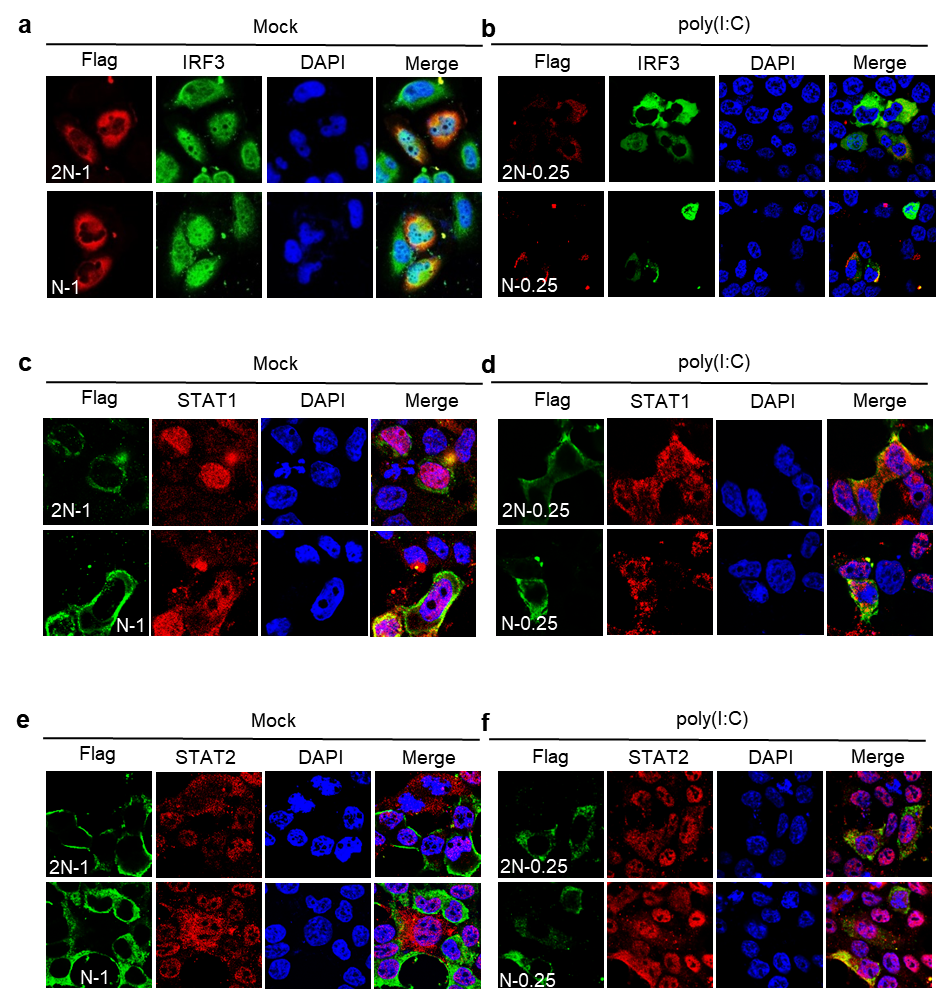
**

**Supplementary Figure 4. N proteins dually regulate nuclear translocation of IRF3, STAT1 and STAT2. a** HepG2 cells were co-transfected with GFP-tagged IRF3 and 1 μg GST-tagged SARS-CoV-2 or SARS-CoV N (GST-2N or N) plasmids in 24-well plate. At 24 hpt, cells were subjected to immunofluorescence with anti-GST antibody. **b** HEK293T cells were co-transfected with GFP-tagged IRF3 and 0.25 μg GST-2N or N plasmids in 24-well plate, together with poly(I:C). At 24 hpt, cells were subjected to immunofluorescence. Green: IRF3 signal; Red: N protein signal; Blue: DAPI (4, 6-diamino-2-phenyl indole, nuclei staining). Merge indicate the merged red, green, and blue channels. **c, e** HEK293T cells were co-transfected with HA-tagged STAT1 (**c**) or STAT2 (**e**) and 1 μg GST-2N or N plasmids in 24-well plate. At 24 hpt, cells were subjected to immunofluorescence with anti-GST and HA antibodies. **d, f** HEK293T cells were co-transfected with HA-tagged STAT1 (**d**) or STAT2 (**f**) and 0.25 μg GST-2N or N plasmids, together with poly(I:C). At 24 hpt, cells were subjected to immunofluorescence. Green: N protein signal; Red: STAT signal; Blue: DAPI. Merge indicate the merged red, green, and blue channels.

Figure. S5.

**
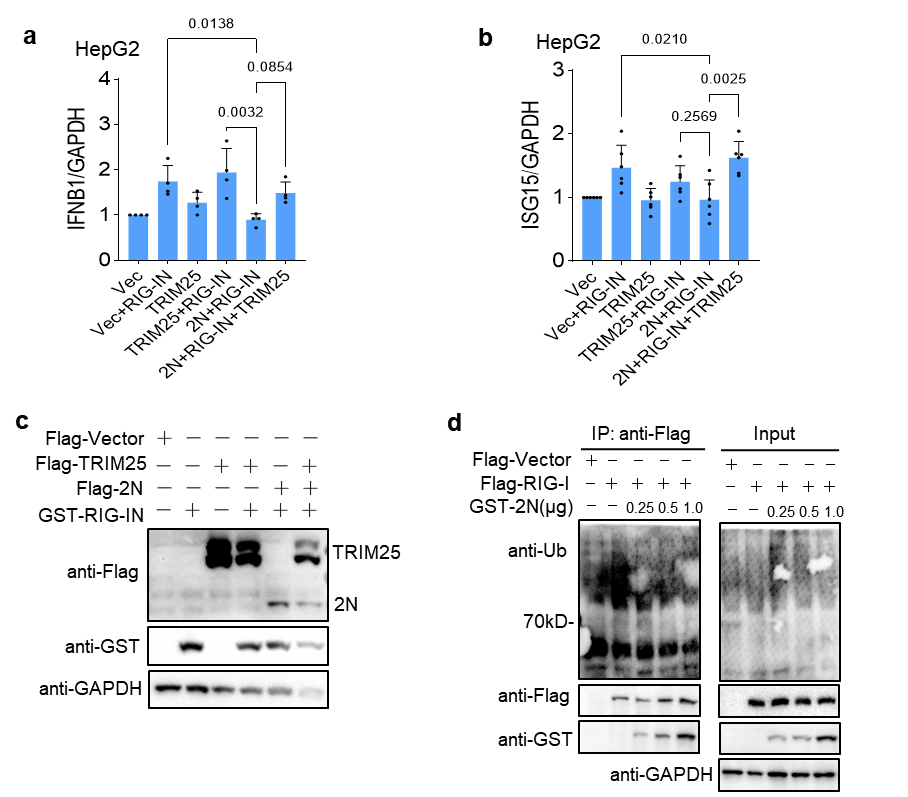
**

**Supplementary Figure 5. Low-dose 2N protein inhibits IFN-I production through TRIM25. a-c** HepG2 cells were co-transfected with the plasmids as indicated. At 24 hpt, qPCR were conducted to detect *IFNB1* and *ISG15* expression (**a, b**), and the transfected protein was analyzed by immunoblot (**c**). **d** HEK293T cells in 6-well plate were transfected with the plasmids as indicated. After 24 h, anti-Flag immunoprecipitates were analyzed by immunoblot with anti-Ub, Flag and GST antibodies.

Figure. S6.

**
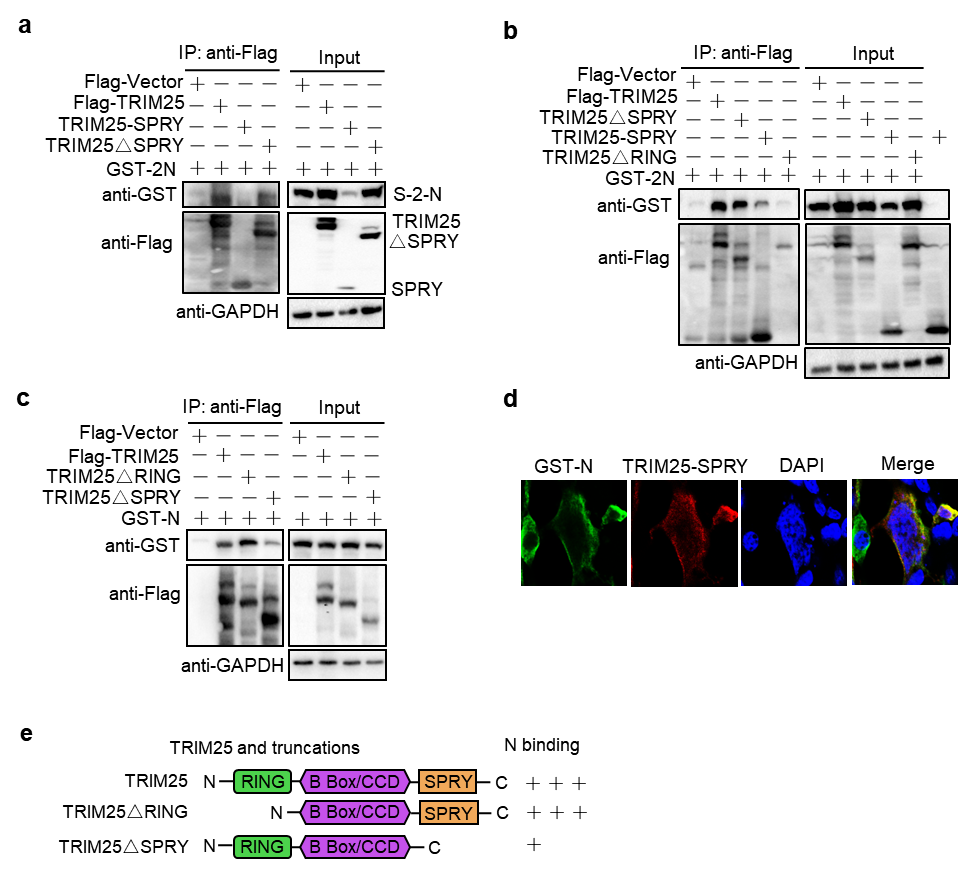
**

**Supplementary Figure 6. The interaction domains of the SARS-CoV-2 N protein and TRIM25. a, b** HEK293T cells were transfected with the indicated plasmids expressing full-length or truncated TRIM25, together with GST-SARS-CoV-2 N (GST-2N). After 24 h, anti-Flag immunoprecipitates were analyzed by immunoblot with anti-GST and Flag antibodies. **c** HEK293T cells were transfected with the indicated plasmids, together with GST-SARS-CoV N (GST-N). After 24 h, anti-Flag immunoprecipitates were analyzed by immunoblot with anti-GST and Flag antibodies. **d** HEK293T cells were transfected with the GST-N and Flag-TRIM25-SPRY domain plasmids. At 24 hpt, the cell were fixed with 4% paraformaldehyde, then incubated with anti-GST and Flag antibodies and subjected to immunofluorescence analyses. Green: N signal; Red: TRIM25-SPRY signal; Blue: DAPI. **e** Domain mapping of the SARS-CoV N and TRIM25 association.

Figure. S7.


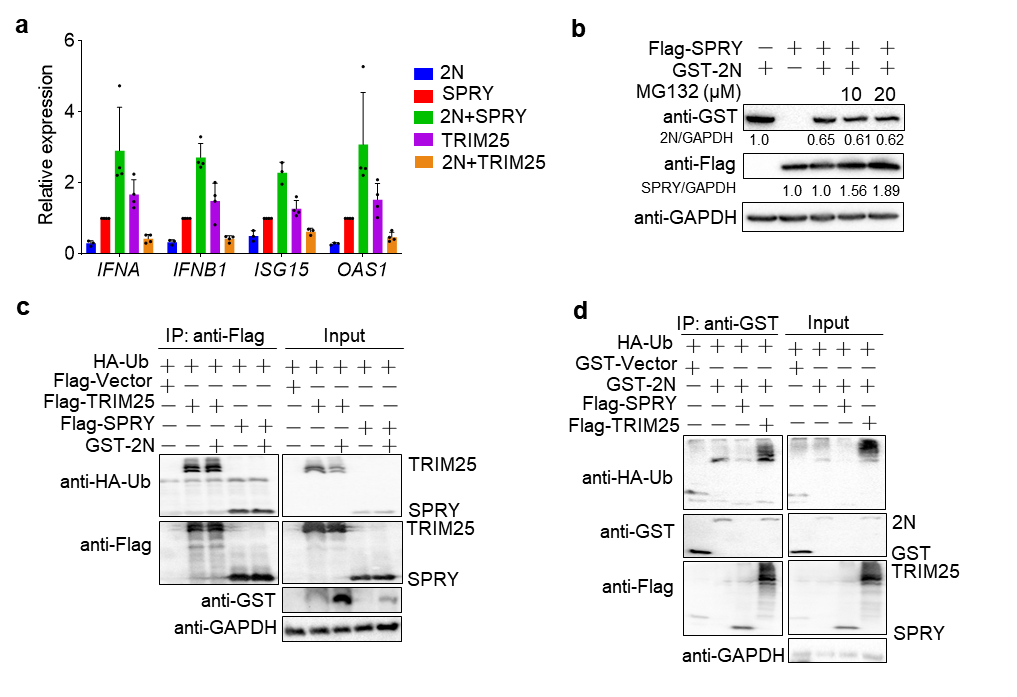


**Supplementary Figure 7. Non-ubiquitination degradation of both SARS-CoV-2 N and SPRY domain after co-transfection.** **a** HEK293T cells were transfected with the plasmids expressing TRIM25 or SPRY domain, together with or without 2N. At 24 hpt, qPCR examined gene expression of the *IFNA*, *IFNB1*, *ISG15* and *OAS1.* **b** HEK293T cells were transfected with the indicated plasmids, together with different doses of MG132, immunoblot analyzed with anti-GST and Flag antibodies. **c** HEK293T cells were transfected with the indicated plasmids, together with or without SARS-CoV-2 N (GST-2N). At 24 hpt, anti-Flag immunoprecipitates were analyzed by immunoblot with anti-HA, Flag and GST antibodies. **d** HEK293T cells were transfected with the indicated plasmids. After 24 h, the cell lysates were incubated using glutathione agarose to purify GST-2N protein. The purified proteins were analyzed by immunoblot with anti-HA, GST and Flag antibodies. Results shown are the mean±SD of at least three independent experiments.

Figure. S8.


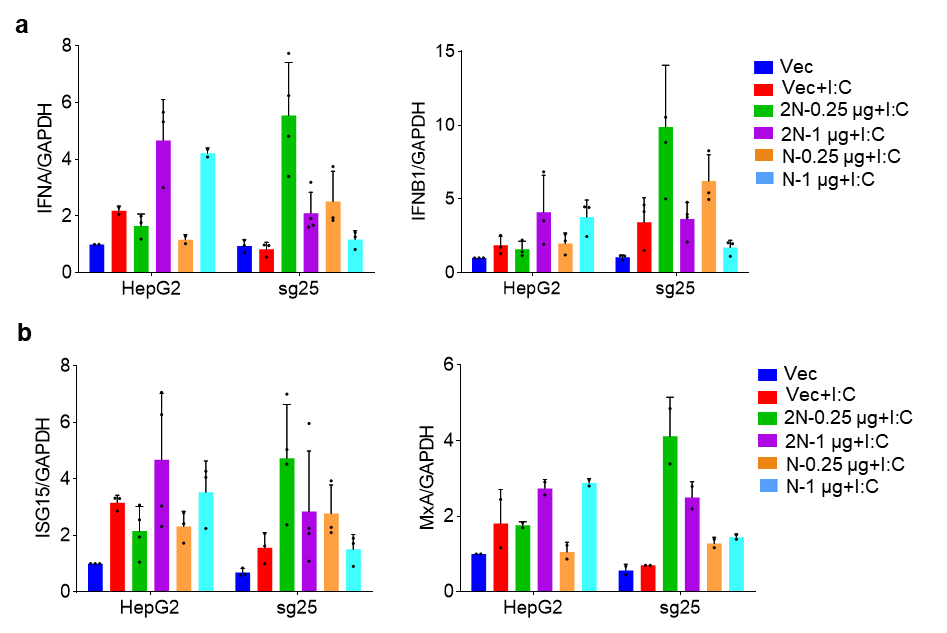


**Supplementary Figure 8. High-dose SARS-CoV-2 N protein promotes IFN-I pathway through TRIM25.** **a, b** TRIM25 knockout (sg25) and WT HepG2 cells were co-transfected with SARS-CoV-2 or SARS-CoV N (2N or N), together with or without poly(I:C). At 24 hpt, the mRNA levels of *IFNA* and *IFNB1* (**a**), *ISG15* and *MxA* (**b**) were determined using qPCR. Results shown are the mean ± SD of at least three independent experiments.

Figure. S9.


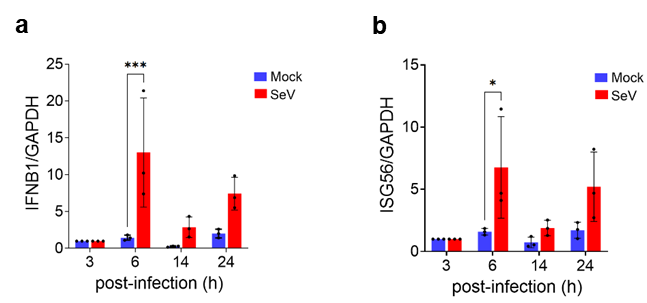


**Supplementary Figure 9. SeV induces IFN-I production in early stage of infection.** **a, b** HEK293T cells were infected with SeV. At 3, 6, 14, and 24 hpi, total RNA was extracted, and the mRNA expression of *IFNB1* (**a**) and *ISG15* (**b**) relative to GAPDH control were examined using qPCR.

Figure. S10.

**
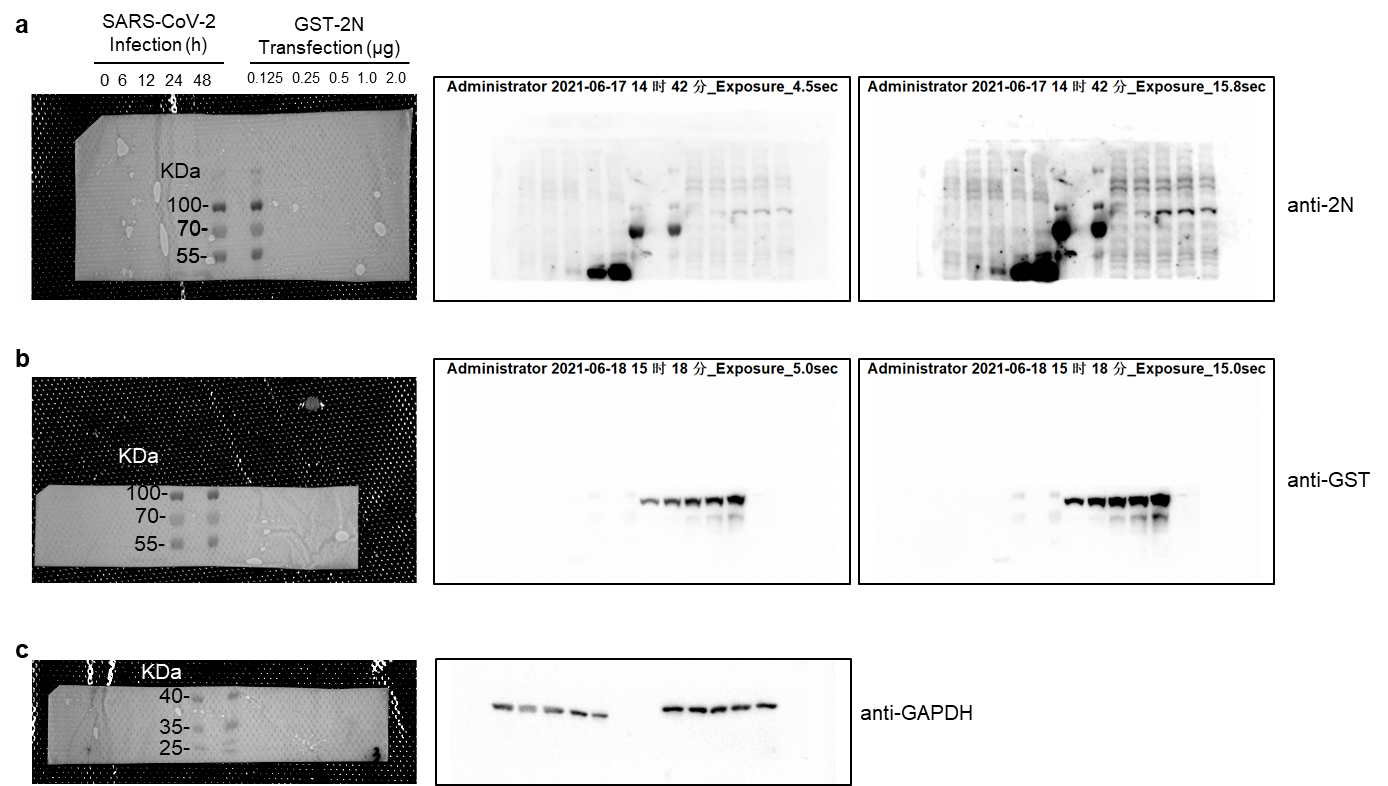
**

**Supplementary Figure 10. Expression of 2N in SARS-CoV-2 infected cells was comparable with 2N transfected cells.** **a-c** Caco-2 cells were infected with SARS-CoV-2 at MOI of 0.01, at 6, 12, 24, and 48 hpi, cells were collected; HEK293T cells were transfected with the indicated GST-tagged 2N plasmid, at 24 hpt, cells were collected; The lysis of infected and transfected cells (50 μg/sample) were analyzed by immunoblot with anti-SARS-CoV-2 N (**a**), anti-GST (**b**) and anti-GAPDH (**c**) antibodies.

Table S1.

**Table S1 Primer pairs used for quantitative real-time PCR (qPCR)**

| **Genes** | **Sequences** | |
| --- | --- | --- |
|  | **Forward (5’ to 3’)** | **Reverse (5’ to 3’)** |
| *IFNA* | GCCTCGCCCTTTGCTTTACT | GGATCAGCTCATGGAGGACAGA |
| *IFNB1* | ATGACCAACAAGTGTCTCCTCC | GGAATCCAAGCAAGTTGTAGCTC |
| *ISG15* | TGGACAAATGCGACGAACC | CCCGCTCACTTGCTGCTT |
| *OAS1* | GGCAGAAATCACAGCAAG | GAGGGAGGGAGTTCACAT |
| *MxA* | CCAGGACTACGAGATTGAG | CAGCCACTCTGGTTATGC |
| *GAPDH* | GAGTCAACGGATTTGGTCGT | TGGGATTTCCATTGATGACA |
| *SPRY-1* | CACTACTGGGAGGTGGAGC | GGTGGAGGGCAGGGTTTT |
| *SPRY-2* | AAACCCTGCCCTCCACCA | GCCTCAGTAAAGTCCACCCT |
| *SARS-2-N-1* | TCTACGCAGAAGGGAGCA | AGCAAGAGCAGCATCACC |
| *SARS-2-N-2* | AACATTCCCACCAACAGA | TGCAGCAGGAAGAAGAGT |
| *IL1B* | ACAGTGGCAATGAGGATG | TGTAGTGGTGGTCGGAGA |
| *IL6* | CCTGACCCAACCACA | CTACATTTGCCGAAGAG |
| *TNFA* | CAAGCCCTGGTATGAGC | GCAATGATCCCAAAGTAGA |
